# Tense production in French-speaking participants with Alzheimer's disease: What about discourse? Contribution of a storytelling-in-sequence task

**DOI:** 10.1177/13872877251376028

**Published:** 2025-09-09

**Authors:** Evodie Schaffner, Mélanie Sandoz, Pauline Chatton, Katia Iglesias, Natacha Cordonier, Gilles Allali, Marion Fossard

**Affiliations:** 1 Institut des Sciences logopédiques, Faculté des Lettres et Sciences Humaines, 27214 University of Neuchâtel, Neuchâtel, Switzerland; 2 Service Universitaire de Neuroréhabilitation, 30635 Centre Hospitalier Universitaire Vaudois, Lausanne, Switzerland; 3 School of Health Sciences (HEdS-FR), 128872 HES-SO University of Applied Sciences and Arts Western Switzerland, Fribourg, Switzerland; 4 Brain and Mind Centre, School of Psychology, Faculty of Science, 27214 University of Sydney, Sydney, Australia; 5 Centre Leenaards de la Mémoire, 30635 Centre Hospitalier Universitaire Vaudois, Lausanne, Switzerland; 6 Center for Research in Neuroscience, Faculty of Biology and Medicine, University of Lausanne, Lausanne, Switzerland

**Keywords:** Alzheimer's disease, language, memory, time

## Abstract

**Background:**

The production of verbal tenses is impaired in people with Alzheimer's disease (AD), as shown by several studies focusing on time reference and using sentence completion tasks. However, there is currently a limited understanding of how tense is produced in discourse with this disease. Discourse is interesting as it involves building a mental representation of the event to be narrated with its temporal framework and translating this framework into language using tense. A shift from the present perspective to the time of the event is required. This may be particularly difficult for people with AD, who also show an alteration in mental time travel capacity, which is linked to episodic memory, semantic memory and temporality.

**Objective:**

This study has two aims: (1) to investigate the influence of the temporal framework on tense production in French-speaking people with AD, and (2) to determine whether abilities in temporality, episodic memory and semantic memory predict tense marking in discourse among this population.

**Methods:**

Tense production was assessed using a storytelling-in-sequence task administered to 21 people with biologically probable AD (age: 71.95; % female: 57) and a control group.

**Results:**

The results showed that people with AD produced more verb forms in the present tense in the non-present conditions than in the control group, especially in the future condition. Temporality and semantic memory predict future tense production in this disease.

**Conclusions:**

This study suggests that studying time reference in AD provides information about the ability to mentally travel through time in this population.

## Introduction

Telling stories is a frequent human activity. These stories can take many forms and aim to achieve many different goals, but all of them have a temporal framework that helps the listeners to understand them and construct mental representations of the actions depicted.^1,2^ Temporal anchoring is mainly achieved through time reference, i.e., the linguistic expression of time, which enables us to express concepts of past, present, and future associated with every human experience. In tensed languages like English or French, time reference uses verbal tense to situate events on a timeline. Indeed, tense provides temporal information linking the reported event to a temporal anchoring point.^3^ It therefore situates the moment of any event in relation to the reported event (i.e., as occurring before, during, or after the time of speech corresponding respectively to a past, present, or future event).^4,5^ More specifically, in French, a morphologically complex language, time reference is primarily marked through tense and temporal adverbs (e.g., hier, aujourd’hui, demain; yesterday, today, tomorrow). Tense is encoded through the addition of grammatical morphemes to the verb stem. For instance, in *je marche* (present)*, je marchais* (past), and *je marcherai* (future) (as in other languages, the future tense in French could be defined as a modality, although this view is still debated^6,7^); the morphological variations of the verb *marcher* (to walk) indicate different time references.

Despite its importance in efficient communication, time reference is frequently impaired in several neurodegenerative diseases, notably in Alzheimer's disease (AD). Previous studies in Greek and Italian have demonstrated that people with AD experience difficulties in producing the appropriate tense to complete a sentence.^8,9^ In French, subtle deficits have also been observed for the past tense in a task requiring the selection of tensed verb forms (Schaffner et al., in revision). Such impairments can significantly disrupt everyday communication, particularly when the constructed temporal framework of a reported event does not align with the time of occurrence of that event.

These findings are based on results obtained in artificial tasks, such as sentence completion tasks, which constrain the participant from producing a single expected response leaving them with limited choice. However, in everyday communication, multiple verb forms may be possible, depending on the intended message and the temporal context of the story being told. Therefore, research adopting a more ecological approach is necessary. Investigating the production of tense through verbal inflection in discourse could yield data that more accurately reflect everyday use of time reference. Such studies could also provide valuable insights into the cognitive processes underlying the production of verbal tenses.

When telling a story, the storyteller moves back and forth in time as time shifts are common in discourse.^2,10^ However, if both the discourse (representing the moment of speech of the reported event) and the mental representation of the story (reflecting the construction of the event's mental representation) are always anchored in the present, the storyteller must cognitively detach from the present to focus on the actual time of the reported event. This process of mental time travel predominantly involves projecting one's mind towards the past or the future^11–13^ and is grounded in autobiographical memory, which encompasses both semantic and episodic components.^14–16^ Additionally, it is closely linked to the concept of temporality - the subjective experience of time, which allows us to think of the past, the present and the future together, although in reality they are mutually exclusive.^11^

Episodic memory, semantic memory and temporality are known to be impaired in people with AD,^15,17–23^ suggesting that these deficits may affect time reference in this population.^24^ In contrast, morphological mechanisms appear to remain relatively intact (Schaffner et al., in revision). If the impairment of mental time travel observed in this disease is related to the alteration of abilities in episodic memory, semantic memory and temporality, it may consequently affect the way in which reported events are conveyed through language. Irish et al.,^25^ for instance, investigated past-tense production in personal event narratives using the Autobiographical Interview.^26^ Their findings showed that people with AD have difficulty in producing past tense forms, instead relying more frequently on the present tense compared to control participants. According to the authors, this reduced temporal specificity in discourse of people with AD arises from a shift in the representation of past personal events, which become increasingly semantic rather than episodic - reflecting the core alteration of episodic memory characteristic of people with AD.^21^ Despite these important findings, the relationship between mental time travel and verbal inflection remains underexplored. To our knowledge, and based on a recent systematic review,^24^ no subsequent study has specifically investigated how mental time travel difficulties influence the linguistic encoding of events’ temporal framework in discourse through verbal inflection. Given the well-documented impairments in mental time travel in people with AD, one would expect significant difficulties in the production of both past and future tense forms.

When referring to a past or future event, the time of the event does not align with the moment of its utterance. This non-alignment requires the speaker to perform a time shift from the present to the past or future perspective to accurately produce a past or future tense, making this production difficult. The difficulty caused by this temporal misalignment has been specifically theorized in relation to past-tense production through the PAst DIscourse LInking Hypothesis (PADILIH).^27^

Given that the capacity for mental time travel may influence how events are linguistically encoded - particularly through tense production - a memory-based perspective also warrants consideration. From this perspective, shifting from a present to a past perspective may be particularly costly, as constructing a mental representation of a past event relies on retrieving information from past personal experiences as well as accessing relevant semantic information knowledge.^28–30^

Regarding the future, research on mental time travel has shown that episodic future thinking - the ability to mentally travel into the future - is also impaired in people with AD.^17,31^ Interestingly, these studies also found that some cognitive processes involved in mental travel into the past are also involved in episodic future thinking.^32–35^ Building a mental representation of a future event also calls for episodic and semantic content. Since future events do not yet exist, they must be built from personal experience, drawing on episodic content of past events. Additionally, semantic information, linked to general scenarios, is essential for forming coherent mental representations of future events.^32,35,36^ Moreover, generating a future event is also more demanding in terms of detail recombination compared to the construction of a past event.^32,37^ Reporting future events may therefore be particularly challenging, as it involves two key steps: retrieving relevant past information and integrating it into familiar, coherent scenarios, while situating it within a future temporal framework.

Translating the temporal framework of a reported event into the appropriate tense therefore constitutes a particularly complex process, in which mental time travel and the production of time reference (i.e., tense) may interact. Further studies, extending beyond the sentence level and using discursive tasks are required to gain deeper insights into the mechanisms underlying this translation process in people with AD. Indeed, people with AD usually encounter difficulty in mental time travel due to impairments of episodic memory, semantic memory and temporality. Discourse offers the opportunity to look at the past or the future from the present perspective while requiring the production of the appropriate tense to convey the intended temporal framework. Using discourse-based tasks, the present study investigates whether difficulties in mental time travel influence the production of time reference in people with AD, addressing two objectives:

1. To investigate the influence of the temporal framework on the production of tenses by French-speaking people with AD. In other words, compared to control participants, how do people with AD produce tenses in a story, according to a given temporal framework? We expect participants with AD to have difficulties in accurately marking tense corresponding to a given temporal framework compared to control participants. Specifically, we expect that participants with AD would produce more present tenses than control participants according to the temporal framework, especially in the non-present conditions, suggesting difficulty to travel mentally in time.^1,28,30,33,35^
2. To determine whether abilities in temporality, episodic memory, and semantic memory predict tense marking in discourse in people with AD. We expect time reference performance of participants with AD to be predicted by abilities involved in mental time travel. More specifically, episodic memory, semantic memory, and temporal processing (i.e., temporality) are expected to play a role in tense marking, particularly in the production of past and future tenses, which require greater reliance on mental time travel.^19,25,38^

## Methods

### Participants

Two groups of participants took part in the study. Twenty-one participants with a diagnosis of AD confirmed by cerebrospinal fluid (CSF) or amyloid PET biomarkers were included in the AD group and twenty-one healthy participants matched in age, gender, and education to the AD group were included in the control group (see Table 1), resulting in a total of 42 participants. All participants are the same as those included in Schaffner et al. (in revision).

**Table 1.** Sociodemographic information and performance on cognitive testing in the AD group and the control group.

	Task	Alzheimer (n = 21)		Control group (n = 21)		Non-parametric tests	
*Demographic*							
Gender		12F : 9M		11F : 10M		NA	NA
		*Mean*	*SD*	*Mean*	*SD*	*U*	*p*
Age		71.95	9.09	70.81	8.96	202	0.64
Education		13.48	2.44	13.43	2.42	217	0.93
*Language*							
General language functioning	*DTLA* ^a^ *(/100)*	87.89	12.32	97.90	3.83	74.50	**<0**.**001**
Naming	*GREMOTs* ^b^ *(/36)*	31.39	3.38	34.48	2.60	92.00	**<0**.**001**
Word repetition	*GREMOTs* ^b^ *(/10)*	9.78	0.55	9.95	0.22	188.00	0.27
Verbal inflection	*BEPS* ^c^ *(/24)*	20.28	3.75	23.38	0.97	84.00	**<0**.**001**
Verb fluency	*GREMOTs* ^b^	26.83	10.07	38.76	11.95	99.00	**<0**.**01**
*Cognitive functioning*							
General cognitive functioning	*MoCA* ^d^ *(/30)*	20.33	3.81	28.05	1.56	6.5	**<0**.**001**
Episodic memory	*RL/RI 16 Delayed free recall* ^e^ *(/16)*	3.70*	3.06*	11.81	2.79	6.00	**<0**.**001**
Semantic memory	*CCT* ^f^ *(/32)*	27.52	2.86	30.90	1.09	56.5	**<0**.**001**
Temporality	*Questionnaire of temporality (/18)* ^g^	12.48	2.75	16.71	1.10	28	**<0**.**001**

^a^ Macoir et al.;^39^

^b^ Bézy et al.;^40^

^c^ Coulombe et al.;^41^

^d^ Nasreddine et al.;^42^

^e^ Van der Linden et al.;^43^

^f^ Moore et al.;^44^

^g^ This questionnaire is currently being validated;^45^

SD: standard deviation; U: U of Mann-Whitney; F: female; M: male; NA: nonapplicable; * for this measure n = 10 in the AD group.

All participants were native French speakers or had a very good command of French and normal or corrected vision and hearing. They presented neither a history of psychiatric disorders as described by the DSM-5,^46^ nor symptoms of alcohol or drug dependence disorder according to the DSM-5 criteria, nor focal intracerebral lesions of a post-traumatic or cerebrovascular nature.

Participants of the AD group were recruited from the Centre Leenaards de la Mémoire of the Centre Hospitalier Universitaire Vaudois (CLM-CHUV) in French-speaking Switzerland. They had to show biomarkers linked to the AD diagnostic according to the ATN classification,^47^ be diagnosed with probable AD according to the revised NINCDS-ADRDA criteria,^21^ have a global Clinical Dementia Rate (CDR) score of 0.5 to 1, and show mild to moderate cognitive impairment according to the Montreal Cognitive Assessment (MoCA) score.^42^

Participants of the control group had to show preserved global cognitive functioning by obtaining a score equal to or greater than 26/30 on the MoCA^42^ and a score equal to or greater than the threshold scores stratified for age and educational level on the French language screening test of “Dépistage des Troubles du Langage chez l'Adulte et la personne âgée” (DTLA).^39^

The participants in this study were involved in a larger research project on time reference in French. The local ethics committee (Commission cantonale d’éthique de la recherche sur l’être humain du Canton de Vaud – CER-VD) approved the study on 9 March 2021 (approval no. 2020-02723). All participants gave their written consent after having been informed orally and in writing of the study's objectives.

Table 1 shows sociodemographic information of the participants and their performance on the tests assessing cognitive functions. Based on the data distribution, non-parametric Mann-Whitney tests were performed using IBM SPSS Statistics^48^ to assess whether both groups matched in age and education level, and to compare the performance of both groups on tests assessing temporality, episodic memory and semantic memory.

### Materials and procedure

#### The storytelling-in-sequence task

**Figure 1.**
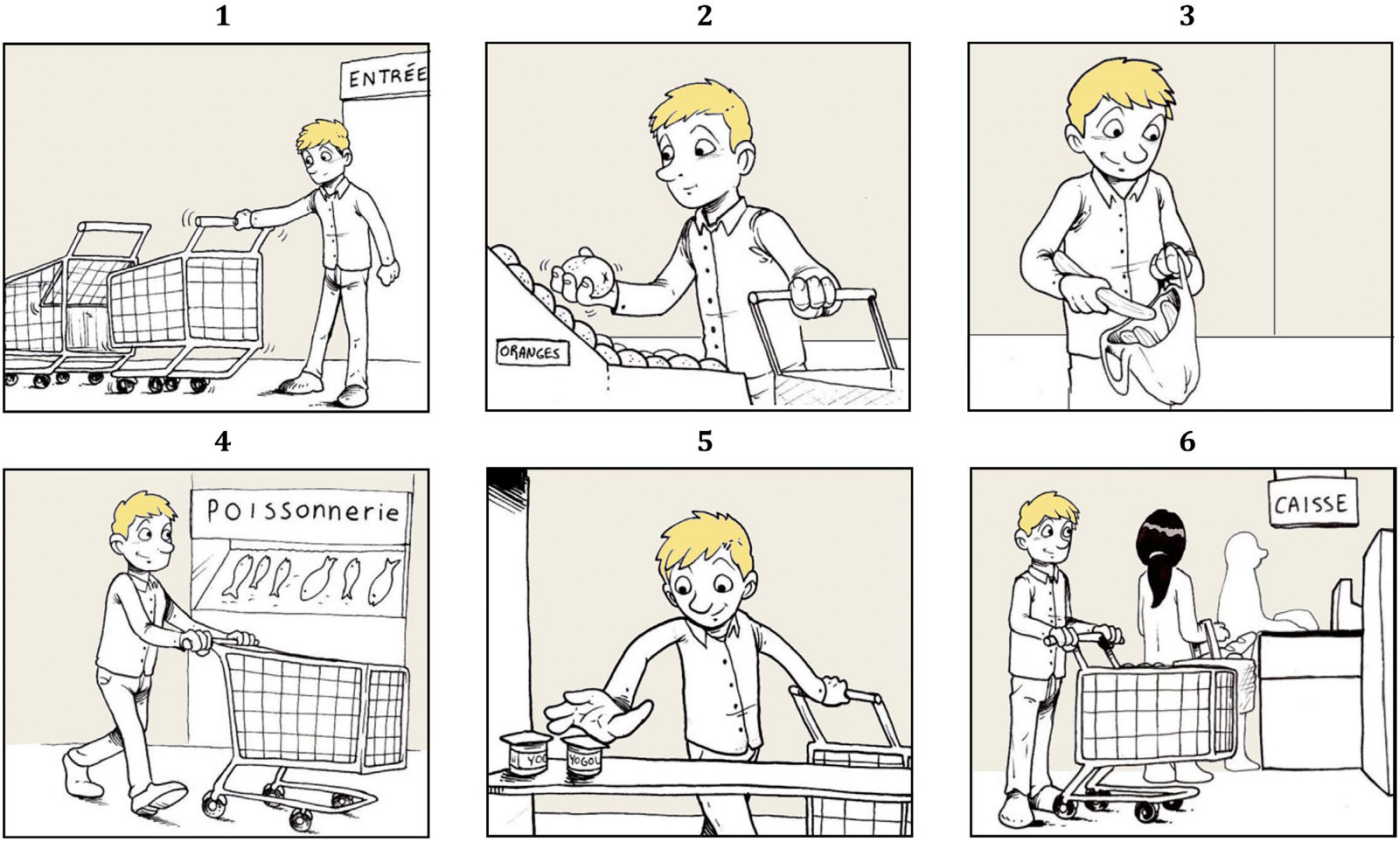
Example of a narrative sequence.

A narrative task corresponding to a storytelling-in-sequence task was used to assess time reference in a discursive context. Six narrative sequences of six colored pictures (10 × 11.5 cm), taken from the study of Fossard et al.,^49^ were used. In these sequences, one or two characters perform everyday activities, such as going to the supermarket or planting flowers for instance (see Figure 1 for an example). Each sequence was presented individually to the participant on an A3 sheet of paper with a priming sentence posing the temporal framework of the story to tell (see Table 2 for the priming sentences used). Out of the six narrative sequences, two stories had to be told by temporal framework (i.e., two for the past, two for the present and two for the future). The same stories were assigned the same temporal frameworks for all participants (see Table 2).

**Table 2.** Priming sentences by temporal framework.

Temporal framework	Priming sentences
Past	***Sequence 1*** : La semaine dernière, Pierre est allé faire ses courses au supermarché. Son frigo était vide. *Last week, Pierre went shopping at the supermarket. His fridge was empty.* ***Sequence 2*** : L'été passé, Marc et Julie ont donné un spectacle de cirque. Ils savaient bien jongler. *Last summer, Marc and Julie put on a circus show. They were good at juggling.*
Present	***Sequence 3*** : Jeanne aime bricoler. Elle prépare une cabane pour les oiseaux. *Jeanne likes to make things. She is making a birdhouse.* ***Sequence 4*** : Anne s’achète de nouvelles chaussures. La vendeuse la conseille. *Anne buys new shoes. The saleswoman advises her.*
Future	***Sequence 5*** : Le week-end prochain, Jacques aura du temps. Il plantera des fleurs. *Next weekend, Jacques will have some time on his hands. He will be planting flowers.* ***Sequence 6*** : La semaine prochaine, Emile et Marie partiront en vacances. Ils feront du camping. *Next week, Emile and Marie will go on vacation. They will go camping.*

For each narrative sequence, the A3 paper and the written priming sentence were presented to the participant. They were informed that the story happened in a certain temporal framework (past, present, or future) and that they had to continue the story starting with the priming sentence. After having carefully looked at the pictures and hearing the priming sentence read aloud by the experimenter, they were asked to tell the story depicted in the narrative sequence.

Each participant had six stories to tell. Both stories of a same time condition were successively told before stories of another time condition were proposed. As stories at the future condition were deemed to be the most difficult to produce, this time condition was never the first one proposed. Indeed, telling stories situated in the future requires not only retrieving past information, but also constructing plausible general scenarios, making the future condition particularly challenging. The order of the time conditions was randomized between participants. No time limit was imposed to participants for telling stories and no feedback was given during the task. The experimenter clearly stated the temporal framework of the coming story (e.g., here, it is a story happening in the present / the past / the future). If a participant did not use the appropriate tense corresponding to the temporal framework, the experimenter waited until the participant had completed the narrative before repeating the instructions (in French, as in other languages, a future event can be referenced by using a present-tense verb form following a future-referring adverb. The task was specifically designed to minimize this alternative use of the future tense). This procedure ensured that any deviation from the expected tense use was not due to misunderstanding or forgetting the task instructions.

All narrations were audio recorded and orthographically transcribed verbatim. Transcriptions were divided (with double slashes) into six parts, corresponding to the six pictures of the narrative sequences. Then, following the procedure of Saffran et al.^50^ for analyzing agrammatic production, all participants’ commentaries on the task or the picture (e.g., what is that ?, I don’t really see what is going on), cancelled words, repetitions or autocorrections were removed (only the last production was considered). The cleaned verbatims were then transferred to a spreadsheet where each verb form was scored. All possible verb tenses in French were listed in the spreadsheet (i.e., one tense per column, e.g., for past tenses: imparfait, passé composé, passé simple, etc.) and the number of verb forms corresponding to the tenses was calculated. For each narration, this table provided the number of words produced by the participant, the number of verbs produced for each tense (inflected and non-inflected verb forms), and the ratios of verb forms produced as a function of tense produced^51^ (i.e., sum of verb forms produced in one tense (e.g., past tense) divided by the total sum of verb forms produced in the story). Two independent experimenters rated the narratives produced by the participants in terms of the inflected verb forms produced, resulting in 90% agreement from the AD group and 96% from the control group. Disagreements were resolved through discussion.

As mentioned above, participants in this study were involved in a larger research project on time reference in French. They underwent cognitive and language assessments before performing the storytelling-in-sequence task. Results from the other tasks are not presented in this article. Three sessions of one and a half to two hours were required for the AD group and two sessions of two hours for the control group. Breaks were provided to avoid fatigue effects. Participants were tested individually in a quiet room, at the CLM-CHUV or at home for the AD group and at the University of Neuchâtel or at home for the control group.

#### Assessment of cognitive functions

Participants’ ability to process time was assessed using a newly developed questionnaire of temporality (i.e., time lived by our consciousness)^45^ built for this study. The questionnaire focused on the most studied dimensions of temporality.^19,52–54^ A first part assessed the evaluation of the duration of daily activities (4 items). Participants were asked to indicate the duration of some activities (e.g., How long does it take to tie a shoelace? How much time has passed since we started the exercises?). A second part assessed time orientation (3 items). Participants were asked to give the full date of the day and the season. They also had to provide information about the last time they met with the experimenter (the questionnaire was administered to participants at the study's second session). Finally, a third part assessed the localization of past historical events (4 items for events that occurred in the last decade and 4 items for events that occurred at the end of the twentieth century). Participants had to judge whether a target event took place before or after another event (e.g., Did man first walk on the moon before or after the fall of the Berlin Wall?). All questions were asked verbally. For the third part of the questionnaire, however, a visual aid was provided and participants had to place a label with the event to be assessed on an arrow representing time. This questionnaire is currently being validated and preliminary results showed a good specificity (0.97) and precision (0.9).^45^

Standardized tests were used to assess participants’ other cognitive functions. Language abilities were assessed with the DTLA^39^ for general language functioning, the GREMOTs^40^ for word naming, word repetition and verb fluency, and the BEPS^41^ for verbal inflection. Regarding memory, the RL/RI 16 Delayed free recall^43^ was used to assess episodic memory and semantic memory was assessed using the Camel and Cactus test (CCT).^44^ Results are reported in Table 1 above.

### Data analysis

Data of the storytelling-in-sequence task were analyzed with R^55^ using the number of inflected verb forms produced in a specific tense. Ratios calculated dividing the sum of verb forms produced in one tense (e.g., past tense) by the total sum of verb forms produced in the story were used as descriptive statistics.

Regarding the first objective of the study, generalized linear mixed models (GLMM) with a distribution for count data were used on the number of inflected verb forms produced in a specific tense. A Poisson distribution was used to process count data. However, after checking the dispersion of the data, a negative binomial distribution was used in case of overdispersion. Three dependent variables were extracted from the participants’ productions: the number of inflected verbs produced in the past, in the present and in the future tenses. For each of our three dependent variables, three separate models were performed to assess the effect of our two independent variables: i) the group (AD, control), and ii) the temporal framework of the story (past, present, future). The interaction between these two independent variables was also tested. Participants were systematically included in the models as a random effect,^56^ but not items as we only had six items (i.e., two items per condition). Random slopes for temporal frameworks were also included in the models, but either did not improve the model or the model did not converge. Finally, given the variability observed in the length of participants’ narratives, the number of words produced was also included in the models as a covariable in all models. Following Pinheiro & Bates (2000),^57^ likelihood ratio tests (LRT) were carried out to assess the effect of our independent variables (group, temporal framework and their interaction) and of the random slopes for temporal framework on our three dependent variables (number of verbs inflected in the past, in the present and in the future tenses). Specifically, the LRT compares the fit of two nested models**:** a) a simpler model (e.g., including only random effects) and b) a more complex model (e.g., adding one fixed effect such as “group”). Post-hoc analyses with a Tuckey correction for multiple comparisons were conducted on the interactions that made a significant contribution to the model, using the package “emmeans”.^58^ Betas, standard errors and odds ratios (OR) are reported for each significant effect. The significance level was set at 0.05. The results regarding the first objective are reported on section “Objective 1 - Effect of the temporal framework of the story on tense production”.

Regarding the second objective of the study, we used the scores at the cognitive tasks (i.e., episodic memory, semantic memory, and temporality) as independent variables, in addition to the group. Interactions between group and scores on the cognitive tasks were also tested to investigate whether some effects of a particular cognitive function could be observed depending on the group. We chose to investigate the involvement of specific cognitive functions on the production of each tense produced (past, present, and future) according to the temporal framework of the story. For instance, models assessing the effect of cognitive function on the production of past tenses were conducted only in the narratives that were made in the past framework. Regarding episodic memory, as eleven participants with AD were not able to perform the RL/RI 16 Delayed free recall^43^ because of their episodic memory loss, analyses were performed only on the scores of 10 participants with AD. The results regarding the second objective are reported in the section “Objective 2 - Effect of cognitive scores on tense production”.

## Results

### Objective 1: effect of the temporal framework of the story on tense production

Table 3 shows the mean and standard deviation for ratios of verb forms produced in the past tense depending on the temporal framework of the story.

**Table 3.** Means and standard deviations (SD) for ratios of verb forms produced in the past tense depending of the temporal framework of the story.

Tense	Temporal framework of the story	Alzheimer (n = 21)		Control group (n = 21)	
		Mean	SD	Mean	SD
Past	Past	0.45	0.33	0.66	0 . 28
	Present	0.10	0.13	0.07	0.15
	Future	0.08	0.09	0.02	0.06

Regarding verb forms produced in the **past tense**, results of the likelihood ratio tests revealed no significant effect of the group (*χ*^2^(1) = 1.35, *p* = 0.24), a significant effect of the temporal framework of the story (*χ*^2^(2) = 156.02, *p* < 0.001) and of the interaction between the group and the temporal framework of the story (*χ*^2^(2) = 17.90, *p* < 0.001). The significant effect of the temporal framework of the story showed that the probability of producing a verb form in the past tense was significantly higher when the temporal framework of the story was set in the past compared to when it was set in the present (β = −1.78 (0.15), z = −11.50, OR = 0.17, *p* < 0.001) or in the future (β = −2.20 (0.17), z = −12.77, OR = 0.11, *p* < 0.001).

Regarding the significant interaction effect, decomposition of the within-subject effect of the temporal framework by group showed that, in the control group the probability of producing a verb form at past tense was significantly higher when the temporal framework of the story was set in the past compared when it was set in the present (β = −2.11 (0.17), z = −12.33, OR = 0.12, *p* < 0.001) and in the future (β = −3.33 (0.32), z = −10.24, OR = 0.04, *p* < 0.001). In this group, the probability of producing a verb form in the past tense was also significantly higher when the temporal framework of the study was set in the present compared to when it was set in the future (β = 1.21 (0.36), z = 3.41, OR = 3.36, *p* < 0.001). In the AD group, the probability of producing a verb form in the past tense was higher when the temporal framework of the story was set in the past compared to when it was set in the present (β = −1.32 (0.15), z = −8.49, OR = 0.27, *p* < 0.001) or in the future (β = −1.42 (0.16), z = −9.00, OR = 0.24, *p* < 0.001). There was no significant difference in the likelihood of producing a verb form in the past tense when the story was set in the present or in the future (β = −0.10 (0.19), z = −0.51, OR = 0.91, *p* = 0.61).

Decomposition of the between-subject effect of group by the temporal framework of the story showed that, when the temporal framework of the story was set in the past, the probability of producing a verb form in the past tense was significantly higher in the control group compared to the AD group (β = 0.47 (0.15), z = 3.09, OR = 1.60, *p* < 0.01). When it was set in the present, the probability of producing a verb form in the past tense was not significantly different between the two groups (β = −0.433 (0.24), z = −1.37, OR = 0.72, *p* = 0.17). When it was set in the future, the probability of producing a verb form in the past tense was significantly higher in the AD group than in the control group (β = 1.44 (0.41), z = 0.37, OR = 4.23, *p* < 0.001).

Table 4 shows the mean and standard deviation for ratios of verb forms produced in the present tense depending on the temporal framework of the story.

**Table 4.** Means and standard deviations (SD) for ratios of verb forms produced in the present tense depending of the temporal framework of the story.

Tense	Temporal framework of the story	Alzheimer (n = 21)		Control group (n = 21)	
		Mean	SD	Mean	SD
Present	Past	0.33	0.30	0.12	0.24
	Present	0.65	0.20	0.67	0.22
	Future	0.41	0.31	0.16	0.23

Regarding verb forms produced in the **present tense**, results of the likelihood ratio tests revealed a significant effect of the group (*χ*^2^(1) = 7.62, *p* < 0.01), a significant effect of the temporal framework of the story (*χ*^2^(1) = 62.72, *p* < 0.001), and a significant effect of the interaction between group and the temporal framework of the story (*χ*^2^(2) = 22.13, *p* < 0.001).

The group effect showed that the probability of producing a verb form in the present tense was significantly higher in the AD group compared to the control group (β = 0.37 (0.13), z = 2.79, OR = 1.44, *p* < 0.01). The effect of the temporal framework of the story showed that the probability of producing a verb form in the present tense was significantly higher when the temporal framework of the story was set in the present compared to when it was set in the past (β = −1.31 (0.15), z = −8.90, OR = 0.27, *p* < 0.001) and in the future (β = −1.08 (0.14), z = −7.56, OR = 0.34, *p* < 0.001). No significant difference was found regarding the probability of producing a verb form in present tense when the temporal framework of the story was set in the past or in the future (β = 0.23 (0.15), z = 1.56, OR = 1.26, *p* = 0.26).

Decomposition of the within-subject effect of the temporal framework of the story by group showed that, at the level group, in both groups the probability of producing a verb form in the present tense was significantly higher when the temporal framework of the story was set in the present compared to when it was set in the past (control: β = −1.80 (0.19), z = −9.46, OR = 0.16, *p* < 0.001 ; AD: β = −0.75 (0.15), z = −4.87, OR = 0.47, *p* < 0.001) and in the future (control: β = −1.45 (0.18), z = −8.10, OR = 0.24, *p* < 0.001; AD: β = −0.61 (0.15), z = −4.01, OR = 0.54, *p* < 0.001). In both groups, no significant difference was found regarding the probability of producing a verb form in the present tense when the temporal framework of the story was set in the past or in the future (control: β = −0.36 (0.21), z = −1.69, OR = 0.70, *p* = 0.09; AD: β = 0.13 (0.16), z = 0.85, OR = 1.15, *p* = 0.40).

Regarding the interaction effect, decomposition of the between-subject effect of group by the temporal framework of the story showed that, when the temporal framework is set in the past and in the future, the probability of producing a verb form in the present tense was significantly higher in the AD group compared to the control group (past: β = 1.06 (0.21), z = 4.66, OR = 0.35, *p* < 0.001; future: β = 0.84 (0.22), z = 3.86, OR = 2.31, *p* < 0.001). No significant difference was reported between the two groups for the probability of producing a verb form in the present tense when the temporal framework of the story was set in the present (β = 0.001 (0.18), z = 0.006, OR = 1.00, *p* = 0.99).

Table 5 shows the mean and standard deviation for ratios of verb forms produced in the future tense depending on the temporal framework of the story.

**Table 5.** Means and standard deviations (SD) for ratios of verb forms produced in the future tense depending of the temporal framework of the story.

Tense	Temporal framework of the story	Alzheimer (n = 21)		Control group (n = 21)	
		Mean	SD	Mean	SD
Future	Past	0.01	0.02	0.00	0.02
	Present	0.04	0.08	0.05	0.06
	Future	0.29	0.29	0.56	0.26

Regarding verb forms produced in the **future tense**, results of the likelihood ratio tests revealed the presence of a significant effect of the group (*χ*^2^(1) = 7.18, *p* < 0.01), a significant effect of the temporal framework of the story (*χ*^2^(1) = 11.62, *p* < 0.001), and the absence of an effect of the interaction between the group and the temporal framework of the story (*χ*^2^(1) = 0.98, *p* = 0.32).

The group effect showed that the probability of producing a verb form in the future tense was significantly lower in the AD group than in the control group (β = −0.61 (0.22), z = −2.72, OR = 0.55, *p* *<* 0.01).

The effect of the temporal framework of the story showed that the probability of producing a verb form in the future tense was higher when the temporal framework of the story was set in the future compared to when it was set in the past (β = −4.07 (0.38), z = −10.61, OR = 0.02, *p* *<* 0.0001) and in the present (β = −2.13 (0.15), z = −13.83, OR = 0.12, *p* < 0.001). The probability of producing a verb form in the future tense was also higher when the temporal framework was set in the present than when it was set in the past (β = 1.95 (0.41), z = −4.79, OR = 0.14, *p* < 0.0001).

In summary, participants generally respected the temporal framework of the stories (e.g., they produced more past tense verbs in the past temporal framework). However, participants with AD tended to use more present tense verbs than controls, especially in stories set in the past and future.

### Objective 2: Effect of cognitive scores on tense production

Results of the predictors’ analysis showed that, in both groups, none of the cognitive scores used predict the probability of producing verb forms at **past tense** when the temporal framework of the study was set in the past, or the probability of producing verb forms at **present tense** when the temporal framework of the story was set in the present.

Regarding production of verb forms at **future tense** when the temporal framework of the story is set in the future, the model of the predictor analysis revealed a significant effect of the semantic memory (*χ*^2^(1) = 7.42, *p* < 0.01). In both groups, participants who had better performances in the task assessing semantic memory also had a higher probability of producing verb forms in the future tense when the temporal framework of the story was set in the future. A significant effect of interaction was also found between the group and the total score at the temporality questionnaire (*χ*^2^(1) = 4.57, *p* < 0.05). Decomposition of this effect showed that the effect of the total score at the temporality questionnaire is present only in the AD group (β = 0.16 (0.06), z = 2.57, OR = 1.17, *p* < 0.05). In this group, better performances at the questionnaire of temporality are related to a higher probability of producing verb forms in the future tense when the temporal framework of the story was set in the future.

## Discussion

This study investigated the production of time reference in discourse, especially tense marking, in French-speaking people with a diagnosis of AD confirmed by CSF or amyloid PET biomarkers and healthy participants. Two main objectives were pursued: (1) to investigate the influence of the temporal framework on tense production in French-speaking people with AD, and (2) to determine whether temporality, episodic memory and semantic memory are involved in tense marking in discourse in this population. Using a storytelling-in-sequence task, participants told stories depicted in a sequence of pictures with regard to a temporal framework provided through a priming sentence. Their abilities in temporality were assessed using a questionnaire of temporality built for the present study, and their abilities in episodic memory and semantic memory were assessed with standardized tests.

Regarding objective 1, results showed that, compared to the control group, participants with AD produced a greater proportion of verb forms in the present tense when the temporal framework of the story was set in the past and in the future. The production of a high proportion of verb forms in the present tense, in conditions where this tense is not the most appropriate, makes the discourse of participants with AD less specific than that of the control group, largely due to an unspecified temporal framework. This finding is in line with that of Irish et al. (2016) showing that people with AD tend to report more unspecific information as they have difficulties accessing information linked to past personal events. The time reference deficit in people with AD, supported by a higher proportion of verb forms inflected in the present tense in past and future conditions, thus appears to be characterized by difficulties in establishing a coherent and specific temporal framework in discourse. Although Irish et al. (2016) found this result in the narration of past personal events, the present finding shows that difficulties building a coherent temporal framework are also present in the narration of non-personal events in AD. The underspecific temporal framework established in their narratives could then indicate that these people tend to lose the temporality of the events they recount, which they experience from a present perspective, and have difficulty projecting themselves into a temporal framework distinct from the present moment^33,59–61^ This could then be empirical evidence that they mainly live in the “here and now” and have difficulty to travel mentally in time toward the past or the future.

As mentioned by an anonymous reviewer, the difficulty of participants with AD to leave the ‘here and now’ for another timeframe may also be influenced by the nature of the narrative task and the executive dysfunction commonly observed in people with AD.^23^ Indeed, in the storytelling in sequence task used in this study, all the described actions take place ‘in the present’, and are visually accessible to the participants. Despite the use of priming sentences to establish the time frame of the actions in past and future stories, the working memory deficit of the participants with AD included in this study (Schaffner et al., in revision) may have contributed to an increased use of the present tense in these two conditions. However, participants with AD exhibited distinct patterns of performance in the past and future conditions: while they produced many past-tense verb forms in the past condition, their productions in the future condition were predominantly in the present tense. If the task itself had primarily driven a focus on the depicted actions, we would have expected a similar pattern across both conditions, with an equally high proportion of present-tense verb forms.

This divergence in verb usage was especially pronounced in the future condition, which yielded an unexpected result. Unlike the past condition, where participants used mainly past-tense forms, the future condition was the only one in which most verb forms did not correspond to the temporal framework. Specifically, participants with AD relied more on the present tense (0.41) than on the future tense (0.29), suggesting a distinct difficulty in conceptualizing future events. Such a result could have been more expected in stories with a temporal framework set in the past. Indeed, following the PADILIH,^27^ past tenses should be more impaired than present and future tenses due to the non-alignment between the moment of the event (past) and the moment of speech of this event (present). However, according to this theory, the future would not be particularly altered, since it would be a special case of the present, in which these two moments are aligned, the event having not yet occurred. This conception of the future has since been challenged, particularly by some studies in aphasiology showing that future is not always preserved^62–64^ Explanatory hypothesis regarding the “irrealis” status of future or morphosyntactic proprieties of this tense were proposed to explain these results. Indeed, as future events do not yet exist and are not definite, some authors suggest that future tense owns an “irrealis” dimension.^65^ This dimension could then be a semantic/pragmatic factor affecting the production of this tense, making it more difficult to produce, as has been shown in aphasia.^62,64,66^ With the specific cognitive profile found in people with AD (i.e., the progressive alteration of episodic memory and difficulties in temporality and mental time travel), a more mnesic explanation may be proposed.^18,21,22,33,67^

Several studies focusing on mental time travel showed that building a mental representation of a future event largely relies on episodic memory and semantic memory, as it has already been shown for past events.^31–35,68^ More precisely, building a mental representation of a future event calls for both types of memory. Episodic memory allows to access information related to past events and recombine it to create a coherent mental representation of the future event. Semantic memory provides the necessary information to build plausible scenarios.^32,35,36,69^ Therefore, telling a story using a future temporal framework would be particularly complex and demanding in processing resources. Results related to the second objective of the present study tend to support this hypothesis as the participant scores obtained in semantic memory predict their ability to produce verb forms in the future tense in the future condition. In the AD group, performance on the questionnaire of temporality also predicts the probability of producing future tense when the temporal framework of the story is set in the future.

Furthermore, regarding the second objective, ability in episodic memory, semantic memory and temporality did not predict the production of past and present tenses in a storytelling-in-sequence task. This result could have been expected for present tense as no mental time travel is needed to produce this tense, the moment of the event being simultaneous with the one of utterance of the event. It is therefore more surprising for past tense, given the non-alignment between the moment of the event and the moment of speech of the event that exists for this tense. As episodic memory does not predict the production of future tense either, one possible explanation may lay in tasks used to assess this memory. Indeed, the task used to assess episodic memory (i.e., the RL/RI 16 Delayed free recall^43^) mainly targets verbal anterograde memory, an important facet of episodic memory but not the one most closely associated with mental time travel. Other tasks specifically assessing the ability to mentally travel through episodic memory would then be useful to better explore the potential link between this memory and time reference. For instance, the Autobiographical Interview^26^ could be an interesting discursive task to use, as reporting personal events calls for episodic memory and mental time travel.

The strong influence of semantic memory and temporality on the production of future tense verb forms when the temporal framework of the story is set in the future, as discussed in relation to the first aim of the study, is also relevant to the second aim of the study. This finding is particularly noteworthy, as semantic memory and temporality appear to be involved in future-tense production only when the temporal framework of the story is set in the future. The finding that semantic memory predicts future-tense production but not past-tense production in both populations suggest that semantic information plays a crucial role in constructing general scenarios when reporting future events.^32,35–37,69^

Moreover, the implication of temporality in future-tense production among people with AD suggests a specific difficulty in processing future events in this population. Interestingly, Schaffner et al. (in revision) found that these same participants with AD had no difficulty in producing future-tense verb forms in sentence completion tasks, demonstrating that their ability to inflect verbs in the future tense remains intact. Instead, the difficulties observed in the present study in establishing the appropriate temporal framework for future-oriented narratives likely stem from an impaired ability to mentally represent future events and construct the corresponding temporal perspective. Shifting from the present perspective to narrate a future event seems to be particularly challenging for people with AD. As previously discussed, the future tense is distinct in that its underlying mechanisms are complex and its referential function involves events that have not yet happened.^27,64,65^ In that sense, future events may be inherently difficult to predict. Supporting this notion, a recent study by Xu et al.^70^ showed that predicting a future event is more difficult than retrodicting (i.e., inferring) a past event. Further research is therefore needed to better understand the mechanisms involved in conceptualizing future events and how these processes are translated into language through time reference.

Several limitations of the present study must be acknowledged. First, although the sample size is comparable to or larger than those used in previous studies in this field, and despite the confirmation of AD through CSF or amyloid PET biomarkers, the overall sample remains relatively small. As a result, the findings – particularly those related to objective 2, which involved predictive analyses – should be treated with caution, given the limitations inherent to the statistical methods used. A larger sample size would likely have enhanced the robustness and generalizability of the results. In addition, all participants in the AD group had mild to moderate AD. It might therefore be interesting to study the production of time reference in discourse with disease progression. Secondly, as mentioned above, standardized tasks were used to assess episodic and semantic memory, complemented by predictive analyses to investigate whether these abilities influence the production of time reference in discourse. However, regarding episodic memory, only ten participants were able to complete the episodic memory assessment task. It would then be interesting to use a more natural task in which episodic memory, semantic memory and discourse are intertwined. For example, using an autobiographical interview-type task might offer a deeper understanding of the relationship between time reference and mental time travel.

In conclusion, this study demonstrates that French-speaking people with AD construct stories with a less specific temporal framework than control participants, as evidenced by the production of a higher proportion of verb forms inflected in the present tense in non-present conditions. This finding provides empirical evidence that people with AD mostly live in the “here and now” and experiment difficulties with mental time travel. Moreover, the production of future-tense verb forms in people with AD appears to rely on semantic memory and temporality, making it particularly cognitively costly. Future tense is the only condition in which cognitive function predict the production of inflected verb forms. Further research is needed to better understand the cognitive and linguistic mechanisms involved in future-tense production in people with AD.

## References

[1] Zacks JM Tversky B . Event structure in perception and conception. Psychol Bull 2001; 127: 3–21. 11271755 10.1037/0033-2909.127.1.3

[2] Zwaan RA . Time in language, situation models, and mental simulations. Lang Learn 2008; 58: 13–26.

[3] Klein W . Time in language, language in time. Lang Learn 2008; 58: 1–12.

[4] Grisot C . Cohesion, Coherence and temporal reference from an experimental corpus pragmatics perspective. Cham: Springer International Publishing, 2018.

[5] Reichenbach H . Elements of symbolic logic. New York: McMillan Compagny, 1947.

[6] de Saussure L . Modalité épistémique, évidentialité et dépendance contextuelle. Lang Fr 2012; 173: 131–143.

[7] de Saussure L Morency P . A cognitive-pragmatic view of the French epistemic future. J Fr Lang Stud 2012; 22: 207–223.

[8] Fyndanis V Arfani D Varlokosta S , et al. Morphosyntactic production in Greek- and Italian-speaking individuals with probable Alzheimer’s disease: evidence from subject–verb agreement, tense/time reference, and mood. Aphasiology 2018; 32: 61–87.

[9] Fyndanis V Manouilidou C Koufou E , et al. Agrammatic patterns in Alzheimer’s disease: evidence from tense, agreement, and aspect. Aphasiology 2013; 27: 178–200.

[10] Zwaan RA . Processing narrative time shifts. J Exp Psychol Learn Mem Cogn 1996; 22: 1196–1207.

[11] Conche M . Temps, temporalité, temporalisation. L’enseignement Philos 2009; 59: 9–20.

[12] Mahr JB Greene JD Schacter DL . A long time ago in a galaxy far, far away: how temporal are episodic contents? Conscious Cogn 2021; 96: 103–224. 10.1016/j.concog.2021.103224 PMC8633156 34715457

[13] Mahr JB Schacter DL . Mnemicity versus temporality: distinguishing between components of episodic representations. J Exp Psychol Gen 2022; 151: 2448–2465. 35324239 10.1037/xge0001215 PMC9489600

[14] Addis DR . Mental time travel? A neurocognitive model of event simulation. Rev Philos Psychol 2020; 11: 233–259.

[15] Liu L Bulley A Irish M . Subjective time in dementia: a critical review. Brain Sci 2021; 11: 1502. 34827501 10.3390/brainsci11111502 PMC8616021

[16] Tulving E . Episodic memory: from mind to brain. Annu Rev Psychol 2002; 53: 1–25. 11752477 10.1146/annurev.psych.53.100901.135114

[17] Addis DR Sacchetti DC Ally BA , et al. Episodic simulation of future events is impaired in mild Alzheimer’s disease. Neuropsychologia 2009; 47: 2660–2671. 19497331 10.1016/j.neuropsychologia.2009.05.018 PMC2734895

[18] Carrasco MC Guillem MJ Redolat R . Estimation of short temporal intervals in Alzheimer’s disease. Exp Aging Res 2000; 26: 139–151. 10755220 10.1080/036107300243605

[19] El Haj M Moroni C Samson S , et al. Prospective and retrospective time perception are related to mental time travel: evidence from Alzheimer’s disease. Brain Cogn 2013; 83: 45–51. 23872099 10.1016/j.bandc.2013.06.008

[20] Grober E Hall CB Lipton RB , et al. Memory impairment, executive dysfunction, and intellectual decline in preclinical Alzheimer’s disease. J Int Neuropsychol Soc 2008; 14: 266–278. 18282324 10.1017/S1355617708080302 PMC2763488

[21] McKhann GM Knopman DS Chertkow H , et al. The diagnosis of dementia due to Alzheimer’s disease: recommendations from the National Institute on Aging-Alzheimer’s Association workgroups on diagnostic guidelines for Alzheimer’s disease. Alzheimers Dement 2011; 7: 263–269. 21514250 10.1016/j.jalz.2011.03.005 PMC3312024

[22] Papagno C Allegra A Cardaci M . Time estimation in Alzheimer’s disease and the role of the central executive. Brain Cogn 2004; 54: 18–23. 14733896 10.1016/s0278-2626(03)00237-9

[23] Wilson RS Leurgans SE Boyle PA , et al. Cognitive decline in prodromal Alzheimer disease and mild cognitive impairment. Arch Neurol 2011; 68: 351–356. 21403020 10.1001/archneurol.2011.31 PMC3100533

[24] Schaffner E Sandoz M Grisot C , et al. Mental time travel and time reference difficulties in Alzheimer’s disease: are they related? A systematic review. Front Psychol 2022; 13: 858001. 35615204 10.3389/fpsyg.2022.858001 PMC9126194

[25] Irish M Kamminga J Addis DR , et al. Language of the past’ - exploring past tense disruption during autobiographical narration in neurodegenerative disorders. J Neuropsychol 2016; 10: 295–316. 26014271 10.1111/jnp.12073

[26] Levine B Svoboda E Hay JF , et al. Aging and autobiographical memory: dissociating episodic from semantic retrieval. Psychol Aging 2002; 17: 677–689. 12507363

[27] Bastiaanse R Bamyaci E Hsu C-J , et al. Time reference in agrammatic aphasia: a cross-linguistic study. J Neurolinguistics 2011; 24: 652–673. 26451073 10.1016/j.jneuroling.2011.07.001 PMC4594877

[28] Brunec IK Chadwick MJ Javadi A-H , et al. Chronologically organized structure in autobiographical memory search. Front Psychol 2015; 6: 338. 25859236 10.3389/fpsyg.2015.00338 PMC4373267

[29] Clewett D DuBrow S Davachi L . Transcending time in the brain: how event memories are constructed from experience. Hippocampus 2019; 29: 162–183. 30734391 10.1002/hipo.23074 PMC6629464

[30] Radvansky GA Zacks JM . Event boundaries in memory and cognition. Mem Time Space 2017; 17: 133–140. 10.1016/j.cobeha.2017.08.006 PMC5734104 29270446

[31] El Haj M Antoine P Kapogiannis D . Flexibility decline contributes to similarity of past and future thinking in Alzheimer’s disease. Hippocampus 2015; 25: 1447–1455. 25850800 10.1002/hipo.22465 PMC5460916

[32] Addis DR Schacter DL . Constructive episodic simulation: temporal distance and detail of past and future events modulate hippocampal engagement. Hippocampus 2008; 18: 227–237. 18157862 10.1002/hipo.20405

[33] El Haj M Antoine P Kapogiannis D . Similarity between remembering the past and imagining the future in Alzheimer’s disease: implication of episodic memory. Neuropsychologia 2015; 66: 119–125. 25448861 10.1016/j.neuropsychologia.2014.11.015 PMC5471357

[34] Schacter DL Gaesser B Addis DR . Remembering the past and imagining the future in the elderly. Gerontology 2013; 59: 143–151. 22987157 10.1159/000342198 PMC3645892

[35] Schacter DL Addis DR . The cognitive neuroscience of constructive memory: remembering the past and imagining the future. Philos Trans R Soc B Biol Sci 2007; 362: 773–786. 10.1098/rstb.2007.2087 PMC2429996 17395575

[36] D’Argembeau A . Zooming in and out on one’s life: autobiographical representations at multiple time scales. J Cogn Neurosci 2020; 32: 2037–2055. 32163320 10.1162/jocn_a_01556

[37] Addis DR . Are episodic memories special? On the sameness of remembered and imagined event simulation. J R Soc N Z 2018; 48: 64–88.

[38] Auclair-Ouellet N Macoir J Laforce R , et al. Regularity and beyond: impaired production and comprehension of inflectional morphology in semantic dementia. Brain Lang 2016; 155–156: 1–11. 10.1016/j.bandl.2016.02.002 26994740

[39] Macoir J Fossard M Lefebvre L , et al. Detection test for language impairments in adults and the aged—A new screening test for language impairment associated with neurodegenerative diseases: validation and normative data. Am J Alzheimers Dis Other Demen 2017; 32: 382–392. 28639484 10.1177/1533317517715905 PMC10852687

[40] Bézy C Renard A Pariente J . GRÉMOTS: évaluation du langage dans les pathologies neurodégénératives. Paris: De Boeck Supérieur, 2016.

[41] Coulombe V Fossard M Monetta L . BEPS: development, validation, and normative data of a sentence production test in French. Appl Neuropsychol Adult 2021; 28: 378–390. 31357874 10.1080/23279095.2019.1640699

[42] Nasreddine ZS Phillips NA Bédirian V , et al. The Montreal cognitive assessment, MoCA: a brief screening tool for mild cognitive impairment. J Am Geriatr Soc 2005; 53: 695–699. 15817019 10.1111/j.1532-5415.2005.53221.x

[43] Van der Linden M Coyette F Poitrenaud J , et al. L’épreuve de rappel libre / rappel indice à 16 items (RL/RI-16). In: Van der Linden M Adam S Agnel A , et al. (eds) L’évaluation des troubles de la mémoire: Présentation de quatre tests de mémoire épisodique (avec leur étalonnage). Marseille: Solal, 2004, pp.25–47.

[44] Moore K Convery R Bocchetta M , et al. A modified Camel and Cactus Test detects presymptomatic semantic impairment in genetic frontotemporal dementia within the GENFI cohort. Appl Neuropsychol Adult 2022; 29: 112–119. 32024404 10.1080/23279095.2020.1716357

[45] Chatton P Sandoz M Schaffner E , et al. Comment évaluer rapidement les compétences de temporalité de personnes présentant un trouble neurocognitif ? Présentation d’un nouveau questionnaire.

[46] American Psychiatric Association . Diagnostic and statistical manual of mental disorders. 5th edition. Washington, DC: American Psychiatric Association, 2013.

[47] Jack CR Bennett DA Blennow K , et al. NIA-AA Research framework: toward a biological definition of Alzheimer’s disease. Alzheimers Dement 2018; 14: 535–562. 29653606 10.1016/j.jalz.2018.02.018 PMC5958625

[48] IBM Corp . IBM SPSS Statistics for Windows.

[49] Fossard M Achim AM Rousier-Vercruyssen L , et al. Referential choices in a collaborative storytelling task: discourse stages and referential complexity matter. Front Psychol 2018; 9: 176. 29515493 10.3389/fpsyg.2018.00176 PMC5826302

[50] Saffran EM Berndt RS Schwartz MF . The quantitative analysis of agrammatic production: procedure and data. Brain Lang 1989; 37: 440–479. 2804622 10.1016/0093-934x(89)90030-8

[51] Fossard M Sandoz M Schaffner E , et al. Storytelling-in-sequence task (Version 1.0) [Data set]. UNINE data service. 10.60544/g7xr-ne53 (2025).

[52] Grondin S . Timing and time perception: a review of recent behavioral and neuroscience findings and theoretical directions. Atten Percept Psychophys 2010; 72: 561–582. 20348562 10.3758/APP.72.3.561

[53] Hinault T D’Argembeau A Bowler DM , et al. Time processing in neurological and psychiatric conditions. Neurosci Biobehav Rev 2023; 154: 105430. 37871780 10.1016/j.neubiorev.2023.105430

[54] Rivasseau Jonveaux T Trognon A Batt M , et al. Maladie d’Alzheimer et évaluation de la temporalité de la vie quotidienne: l’estimation de durée est-elle mieux préservée en mémoire située ou déclarative? Ann Méd-Psychol Rev Psychiatr 2013; 171: 238–245.

[55] R Core Team . R: A language and environment for statistical computing, https://www.R-project.org/ (2023).

[56] Baayen RH Davidson DJ Bates DM . Mixed-effects modeling with crossed random effects for subjects and items. Spec Issue Emerg Data Anal 2008; 59: 390–412.

[57] Pinheiro JC Bates DM . Mixed-Effects Models in S and S-PLUS. New York: Springer-Verlag, 2000.

[58] Lenth RV . Least-squares means: the R package lsmeans. J Stat Softw 2016; 69: 1–33.

[59] El Haj M Gallouj K Antoine P . Mental imagery and autobiographical memory in Alzheimer’s disease. Neuropsychology 2019; 33: 609–616. 30896237 10.1037/neu0000521

[60] Irish M . Autobiographical memory in dementia syndromes—an integrative review. WIRES Cogn Sci 2023; 14: e1630. 10.1002/wcs.1630 36239297

[61] Irish M Lawlor BA O’Mara SM , et al. Impaired capacity for autonoetic reliving during autobiographical event recall in mild Alzheimer’s disease. Cortex 2011; 47: 236–249. 20153463 10.1016/j.cortex.2010.01.002

[62] Cordonier N Ericson C Schneider L , et al. Time reference in French-speaking people with fluent and non-fluent aphasia (part II): a cluster analysis. Aphasiology 2024; 39: 1097–1121.

[63] Fyndanis V Arcara G Capasso R , et al. Time reference in nonfluent and fluent aphasia: a cross-linguistic test of the PAst DIscourse LInking Hypothesis. Clin Linguist Phon 2018; 32: 823–843. 29513613 10.1080/02699206.2018.1445291

[64] Koukoulioti V Bastiaanse R . Time reference in aphasia: evidence from Greek. J Neurolinguistics 2020; 53: 100872.

[65] Zagona K . Tense, aspect and modality. In: den Dikken M (eds) The Cambridge handbook of generative syntax. Cambridge: Cambridge University Press Cambridge, 2013, pp.746–792.

[66] Rofes A Bastiaanse R Martínez-Ferreiro S . Conditional and future tense impairment in non-fluent aphasia. Aphasiology 2014; 28: 99–115.

[67] Requena-Komuro M-C Marshall CR Bond RL , et al. Altered time awareness in dementia. Front Neurol 2020; 11: 291. 32373055 10.3389/fneur.2020.00291 PMC7186333

[68] Liu L Roquet D Ahmed RM , et al. Examining prefrontal contributions to past- and future-oriented memory disturbances in daily life in dementia. Cortex 2021; 134: 307–319. 33333361 10.1016/j.cortex.2020.11.003

[69] Irish M Piolino P . Impaired capacity for prospection in the dementias – theoretical and clinical implications. Br J Clin Psychol 2016; 55: 49–68. 26014112 10.1111/bjc.12090

[70] Xu X Zhu Z Zheng X , et al. Temporal asymmetries in inferring unobserved past and future events. Nat Commun 2024; 15: 8502. 39353891 10.1038/s41467-024-52627-5 PMC11445511

[71] Fossard M Schaffner E Sandoz M , et al. Temporality tasks (Version 1.0) [Data set]. UNINE data service. 10.60544/8qex-gb41 (2025).

[72] Fossard M Schaffner E Sandoz M , et al. Language and cognitive tests (Version 1.0) [Data set]. UNINE data service. 10.60544/6nh4-7421 (2025).

